# Punishing Children

**Published:** 1881-10

**Authors:** 


### PUNISHING CHILDREN.

Anna C. Brackett, in the *American Journal of Education*, calls the attention of teachers to the liability of children to be punished or corrected without their clearly knowing why. "They may thus perhaps understand," she adds, "what often seems to them so incomprehensible—why a child who has been rebuked for some disorderly conduct repeats the offense almost immediately, giving the impression of willful and malicious wrong-doing. The same mistake is frequently made in recitations. A pupil's answer is pronounced wrong, and the question passed to another, when he does not know what his error is, and often fancies that it lies in quite a different direction from that in which it really lies. One of the most successful teachers we know, is almost invariably in the habit, after having passed a question and received a correct answer, of asking the pupil who had failed: 'Why did I pass that question?" A few trials of this simple interrogation will soon, we think, convince any teacher of the truth of what we say. The most astonishing misunderstandings are thus continually brought to light, and we become convinced of how double-edged a thing is this language which we use so thoughtlessly and freely."—*Sanitarian.*

